# Micronutrients and Diets in the Treatment of Attention-Deficit/Hyperactivity Disorder: Chances and Pitfalls

**DOI:** 10.3389/fpsyt.2020.00102

**Published:** 2020-02-26

**Authors:** Klaus W. Lange

**Affiliations:** Institute of Psychology, University of Regensburg, Regensburg, Germany

**Keywords:** attention-deficit/hyperactivity disorder, micronutrients, diet, lifestyle, treatment

Attention-deficit/hyperactivity disorder (ADHD) is one of the most common psychiatric diagnoses in childhood and adolescence. It is characterized by age-inappropriate levels of inattention, impulsivity, and hyperactivity and is associated with long-term academic, social, and mental health problems (1, 2). Both pharmacotherapy and behavior therapy yield short-term symptom reduction in individuals with ADHD. Psychostimulants, in particular, have been shown to improve attention and to decrease activity levels in children in the short term. However, their impact on academic performance and quality of life is low (3, 4), and initial symptomatic effects are not usually sustained on long-term follow-up (5, 6). The unproven long-term efficacy of commonly used ADHD drugs, together with concerns in regard to adverse effects of medication, which can be as serious as growth retardation and severe cardiovascular events (7, 8), has led to a search for alternative treatment options.

A range of nutrients have been linked to brain development and functioning, and diet may be a relevant factor in the high incidence and prevalence of mental disorders (9). As early as the 1920s, a possible association between food and hyperkinetic behavior was suggested (10). Children with ADHD and healthy controls appear to have different dietary patterns (11–13). Growing evidence suggests that nutrients, diet, and other lifestyle factors may play a role in the pathophysiology and management of mental disorders (14), including ADHD (15, 16).

Major dietary compounds proposed to be helpful in the treatment of ADHD include micronutrients, such as minerals and vitamins, and polyunsaturated fatty acids (PUFAs). Several studies have demonstrated reduced blood plasma levels of various minerals, such as magnesium, iron, and zinc in children with ADHD at group level, and their supplementation may reduce ADHD symptoms in individuals with respective deficiencies. However, evidence in support of this is lacking (17). The questions of whether vitamin deficiencies are involved in the pathophysiology of ADHD and whether vitamin supplements exert therapeutic effects also remain open (15).

The role of omega-3 PUFAs in the pathophysiology and therapy of ADHD is controversial (15, 18). Since blood levels of docosahexaenoic acid (DHA), eicosapentaenoic acid (EPA) and arachidonic acid (AA) have been found to be significantly decreased in children with ADHD compared to controls, numerous clinical studies have examined the effects of omega-3 PUFA supplementation on ADHD symptoms. A systematic review of meta-analyses of double-blind placebo-controlled trials, in which ADHD symptoms were rated by parents and teachers, concluded that the effect sizes for PUFA supplementation were small (19). The pooling of the negative results of a more recent study with previous findings showed no overall effect of omega-3 PUFAs on ADHD symptoms (20). Accordingly, there is currently little support for the efficacy of omega-3 PUFA supplementation on the core symptoms of ADHD.

Various factors may have influenced the results of studies on micronutrient supplementation in ADHD, *e.g.* heterogeneity of design type, dosage, trial duration, or assessment of response. In the case of PUFAs, the mode of administration (fish or supplements), the type of PUFA employed (omega-3 or omega-6 or combination), and the ratio between DHA, EPA, and AA are likely to be important. An increase in the ratio between blood levels of omega-6 to omega-3 PUFAs has been suggested to be more significant in children with ADHD than the absolute concentrations of either (21, 22). Focusing solely on the supplementation of omega-3 PUFAs may not, therefore, be an adequate treatment approach.

Whether or not individuals with low levels of certain micronutrients are more responsive to dietary supplementation is unknown. It is therefore important to identify subgroups of individuals who are most likely to benefit from micronutrient administration, including those with low baseline status. There could also be a micronutrient threshold status, above which dietary supplementation has little effect. Furthermore, given the high prevalence of comorbid conditions accompanying ADHD, it is probable that significant numbers of participants included in the trials of supplementation of micronutrients may have had co-occurring conditions. Observations in several ADHD studies showed that treatment response varied depending on the participants’ comorbidities (23).

The question of whether micronutrient administration is effective at any time during the life span remains to be investigated. A critical time window for positive supplementation effects to be seen may exist; this has been demonstrated, for example, for the lactating period in a rodent model of chronic omega-3 PUFA deficiency (24). Developmental stage may also play a critical role in humans, and various findings emphasize the role of diet during pregnancy. Poor maternal diet during pregnancy may be associated with ADHD in the offspring. Epigenetic changes evident at birth may underlie the link between the consumption of high-fat and high-sugar processed food and confectionary during pregnancy and symptoms of ADHD in children with conduct problems early in life (25). Furthermore, a prenatal diet with a high ratio of omega-6 to omega-3 PUFAs may influence the risk of the development of (subclinical) ADHD symptoms in the offspring during childhood (26). In addition, maternal dietary patterns during pregnancy (low-level healthy diet, high-level Western diet) were significantly associated with children’s trajectories of high symptoms of hyperactivity-inattention (27). These findings suggest that early prevention of ADHD should specifically target diet during pregnancy and infancy. Future studies should therefore examine whether such interventions can prevent the occurrence of ADHD or reduce the severity of symptoms.

The identification of a role of micronutrients and diet in ADHD is hindered by the ill-defined nature of the disorder and the lack of biological markers underpinning its validity (18). Given that the biological and environmental mechanisms underlying ADHD are multifactorial, heterogeneous, and complex (28), it is naïve to expect a simple one-fits-all solution in regard to micronutrients, diets, or any other therapy.

The high-dosage administration of natural and seemingly healthy micronutrients may carry the risk of unwanted side effects. For example, vitamin E supplementation has shown a trend towards an elevated risk of prostate carcinoma (29), while the intake of selenium was found to elevate the risk of diabetes (30). The supplementation of omega-3 PUFAs, particularly at supra-physiological doses for prolonged periods of time, may also be associated with serious adverse effects. Intervention trials with omega-3 PUFAs have reported no serious adverse reactions at the doses administered (31). However, since an important natural source of omega-3 fatty acids is fish and seafood, contamination with methylmercury, dioxins, and polychlorinated biphenyls may increase the risk for some cancers or may harm unborn children when consumed during pregnancy. Fish oil supplements commonly contain antioxidants and oxidation products of omega-3 PUFAs, both of which may lead to adverse reactions. Omega-3 fatty acids are highly prone to oxidative degradation, and a substantial proportion of omega-3 fish oil preparations have been found to significantly exceed the international voluntary safety recommendations for total oxidation (32). Animal studies have found that oxidized lipid products, such as lipid peroxides, can cause harm, including DNA mutations and cancer (33). The effects of oxidized oils on human health should therefore be examined carefully. Possible adverse consequences of the long-term use of vitamin E added as an antioxidant to fish oil supplements should also be considered, since large-scale trials of *α*-tocopherol supplementation have suggested a link to elevated rates of prostate cancer (34). When similar rates of adverse events are observed in comparisons between omega-3 and placebo groups, this applies to short-term effects only.

Recent investigations, instead of assessing the effects of single micronutrients in children with ADHD, have examined dietary patterns, whole diets and other lifestyle-related factors. The findings of these studies suggest that, rather than focusing on specific micronutrients, the entire diet should be considered. For example, the effects of a diet commonly assumed to be healthy (Mediterranean diet) on children and adolescents with newly diagnosed ADHD were examined in a case–control study (35). Low adherence to the diet was positively associated with an increased likelihood of ADHD diagnosis. However, the study design does not allow any conclusions in regard to cause and effect.

Cross-sectional investigations of the effects of micronutrients and diet usually focus on one or several compounds without regard to other lifestyle factors, such as physical activity or sedentary behavior. Recent studies have demonstrated the interrelationships between diet and lifestyle in ADHD. For example, children with ADHD were found to be almost twice as likely to show a reduced number of healthy behaviors (36). Moreover, primary diagnoses with ADHD were significantly reduced in children with better quality of diet, higher levels of physical activity and less time spent playing video games (37).

The gut microbiome appears to play an important role in the bidirectional communication between gut and brain (gut–brain axis). Gut microbiota composition and function have recently been associated with several mental and neurodevelopmental disorders (38). It has been hypothesized that dysbiosis may also contribute to the clinical phenotypes of ADHD (39, 40). For example, a slight increase in Bifidobacterium in the gut appears to be correlated with diminished neural reward anticipation (41), which has been found to be associated with ADHD (42). Recent findings suggest that broad spectrum micronutrient administration might be a treatment option to modulate Bifidobacterium abundance, which could play a role in the modulation and regulation of ADHD symptoms (43). A further exploration of the role of gut microbiota and dysbiosis in contributing to ADHD symptoms and of microbiota-related interventions, including micronutrient supplementation and whole diet changes, in the management of ADHD is warranted.

The role of food hypersensitivities in ADHD is a promising avenue worthy of further exploration. A systematic review of meta-analyses of double-blind placebo-controlled trials found no convincing evidence of therapeutic efficacy of artificial food color elimination, while the few-foods diet, which excludes many foods and additives, may offer new treatment options (19). The hypothesis of a relationship between food hypersensitivity and ADHD is supported by several studies (44–48). A strictly supervised restricted elimination diet has been demonstrated to be a valuable tool in examining whether ADHD symptoms are induced by individual foods (47). Moreover, in a randomized controlled trial, considerable effects of this diet were observed in an unselected group of children with ADHD (47). A recent open nonblinded pilot study has shown a high level of compliance to an oligoantigenic diet in children with ADHD in an out-patient setting, demonstrating its feasibility^1^. The elimination of individual foods for four weeks showed a response rate of around 60%, with statistically significant, positive effects on attention and behavior at group level and dramatic improvements in some children^1^. In addition, children responding to the diet showed a significantly improved quality of life^1^. The response rate observed by Blazynski and colleagues^1^ confirms the results of previous studies (44, 46, 47). The mechanisms underlying the effects observed on ADHD symptoms require further investigation.

In conclusion, unhealthy dietary patterns may precede a poor nutritional biochemistry status affecting ADHD behaviors, and the management of nutrition and diet should always be considered as an avenue towards improving ADHD symptoms. While several lines of evidence point to a potential role of micronutrients and diets in ADHD, a number of caveats need to be considered before any therapeutic recommendation can be made. Observational studies demonstrating associations between diet and micronutrient levels in people with ADHD and the presence or severity of symptoms do not allow any conclusions on causal relationships, since the preference for certain foods or dietary patterns may be a consequence of ADHD behaviors. While a role of single micronutrients in the treatment of ADHD at group level is not supported by current evidence, therapeutic benefits of supplementation may depend on pretreatment nutrient status and may be confined to individuals with specific micronutrient deficiencies. Potential severe adverse effects of micronutrient supplements need to be considered, especially when administered at supra-physiological doses over extended periods of time. Furthermore, the role of whole diets should be explored. Generally improved lifestyle choices may provide more substantial benefits to children with ADHD than dietary changes alone.

The administration of an oligoantigenic diet with subsequent reintroduction of nutrients provides a more personalized approach to ADHD, which can reveal intolerances or allergies to foods and allows the assessment of their impact in ADHD. The high response rate to an oligoantigenic diet of approximately 60% found in several studies indicates that food intolerances may be important in the pathophysiology of ADHD. This intervention should therefore be considered in all children with ADHD. Individuals responding favorably to the diet should undergo an oral food challenge in order to identify individual food intolerances. Future investigations should use blinded designs and long-term outcome follow-up.

## Author Contributions

The author confirms being the sole contributor of this work and has approved it for publication. 

## Conflict of Interest

The author declares that the research was conducted in the absence of any commercial or financial relationships that could be construed as a potential conflict of interest.

## Footnotes

1 Blazynski N, Schneider-Momm K, Overdick L, Clement C, Rauh R, Clement H-W, et al. Oligoantigenic diet in children with ADHD. *Front Psychiatry* (under review).
